# Supplementary material for: HIVprotI: an integrated web based platform for prediction and design of HIV proteins inhibitors
Source: J Cheminform. 2018 Mar 9;10:12. doi: 10.1186/s13321-018-0266-y (PMC5845081; doi:10.1186/s13321-018-0266-y)
Supplement: Supplementary file 2 — Additional file 2. Source code of HIVProtI web server. [file 13321_2018_266_MOESM2_ESM.zip › HIVprotI_Source-code/hivprotI/weka-3-6-6/doc/weka/core/converters/CSVSaver.html]

CSVSaver


---


|  |  |  |
| --- | --- | --- |
| **Overview** \| **Package** \| **Class** \| **Tree** \| **Deprecated** \| **Index** \| **Help** \| **Weka's home** | |  |
| **PREV CLASS**   **NEXT CLASS** | **FRAMES**    **NO FRAMES**     **All Classes** |  |
| SUMMARY: NESTED \| FIELD \| CONSTR \| METHOD | DETAIL: FIELD \| CONSTR \| METHOD |  |


---


## weka.core.converters Class CSVSaver

```
java.lang.Object
  weka.core.converters.AbstractSaver
      weka.core.converters.AbstractFileSaver
          weka.core.converters.CSVSaver
```

**All Implemented Interfaces:**: java.io.Serializable, CapabilitiesHandler, BatchConverter, FileSourcedConverter, IncrementalConverter, Saver, EnvironmentHandler, OptionHandler, RevisionHandler

---

``` public class CSVSaver extends AbstractFileSaver implements BatchConverter, IncrementalConverter, FileSourcedConverter ```

Writes to a destination that is in csv format


Valid options are:

```
 -i <the input file>
 The input file
```

```
 -o <the output file>
 The output file
```

**Version:**
:   $Revision: 7467 $

**Author:**
:   Stefan Mutter (mutter@cs.waikato.ac.nz)

**See Also:**: `Saver`, Serialized Form

---

**Field Summary**

**Fields inherited from interface weka.core.converters.Saver**

`BATCH, INCREMENTAL, NONE`


**Constructor Summary**

`CSVSaver()`
  
          Constructor


| **Method Summary** | |
| --- | --- |
| `Capabilities` | `getCapabilities()`             Returns the Capabilities of this saver. |
| `java.lang.String` | `getFileDescription()`             Returns a description of the file type. |
| `java.lang.String` | `getRevision()`             Returns the revision string. |
| `java.lang.String` | `globalInfo()`             Returns a string describing this Saver |
| `static void` | `main(java.lang.String[] args)`             Main method. |
| `void` | `resetOptions()`             Resets the Saver |
| `void` | `writeBatch()`             Writes a Batch of instances |
| `void` | `writeIncremental(Instance inst)`             Saves an instances incrementally. |

**Methods inherited from class weka.core.converters.AbstractFileSaver**

`cancel, filePrefix, getFileExtension, getFileExtensions, getOptions, getUseRelativePath, getWriter, listOptions, resetWriter, retrieveDir, retrieveFile, runFileSaver, setDestination, setDestination, setDir, setDirAndPrefix, setEnvironment, setFile, setFilePrefix, setOptions, setUseRelativePath, useRelativePathTipText`

**Methods inherited from class weka.core.converters.AbstractSaver**

`getInstances, getWriteMode, resetStructure, setInstances, setRetrieval, setStructure`

**Methods inherited from class java.lang.Object**

`equals, getClass, hashCode, notify, notifyAll, toString, wait, wait, wait`

**Methods inherited from interface weka.core.converters.FileSourcedConverter**

`getFileExtension, getFileExtensions, getUseRelativePath, retrieveFile, setFile, setUseRelativePath`

**Constructor Detail**

### CSVSaver

```
public CSVSaver()
```

:   Constructor


**Method Detail**

### globalInfo

```
public java.lang.String globalInfo()
```

:   Returns a string describing this Saver

    :   **Returns:**: a description of the Saver suitable for displaying in the explorer/experimenter gui

---


### getFileDescription

```
public java.lang.String getFileDescription()
```

:   Returns a description of the file type.

    :   **Specified by:**: `getFileDescription` in interface `FileSourcedConverter` **Specified by:**: `getFileDescription` in class `AbstractFileSaver`
    :   **Returns:**: a short file description

---


### resetOptions

```
public void resetOptions()
```

:   Resets the Saver

    :   **Overrides:**: `resetOptions` in class `AbstractFileSaver`

---


### getCapabilities

```
public Capabilities getCapabilities()
```

:   Returns the Capabilities of this saver.

    :   **Specified by:**: `getCapabilities` in interface `CapabilitiesHandler` **Overrides:**: `getCapabilities` in class `AbstractSaver`
    :   **Returns:**: the capabilities of this object **See Also:**: `Capabilities`

---


### writeIncremental

```
public void writeIncremental(Instance inst)
                      throws java.io.IOException
```

:   Saves an instances incrementally. Structure has to be set by using the
    setStructure() method or setInstances() method.

    :   **Specified by:**: `writeIncremental` in interface `Saver` **Overrides:**: `writeIncremental` in class `AbstractSaver`
    :   **Parameters:**: `inst` - the instance to save **Throws:**: `java.io.IOException` - throws IOEXception if an instance cannot be saved incrementally.

---


### writeBatch

```
public void writeBatch()
                throws java.io.IOException
```

:   Writes a Batch of instances

    :   **Specified by:**: `writeBatch` in interface `Saver` **Specified by:**: `writeBatch` in class `AbstractSaver`
    :   **Throws:**: `java.io.IOException` - throws IOException if saving in batch mode is not possible

---


### getRevision

```
public java.lang.String getRevision()
```

:   Returns the revision string.

    :   **Specified by:**: `getRevision` in interface `RevisionHandler`
    :   **Returns:**: the revision

---


### main

```
public static void main(java.lang.String[] args)
```

:   Main method.

    :   **Parameters:**: `args` - should contain the options of a Saver.


---


|  |  |  |
| --- | --- | --- |
| **Overview** \| **Package** \| **Class** \| **Tree** \| **Deprecated** \| **Index** \| **Help** \| **Weka's home** | |  |
| **PREV CLASS**   **NEXT CLASS** | **FRAMES**    **NO FRAMES**     **All Classes** |  |
| SUMMARY: NESTED \| FIELD \| CONSTR \| METHOD | DETAIL: FIELD \| CONSTR \| METHOD |  |


---
